# Empatica E4 wristband assessment of probable REM sleep behavior disorder in people with Parkinson’s disease. Results from the DIGI.PARK study

**DOI:** 10.3389/fneur.2026.1720068

**Published:** 2026-01-14

**Authors:** Lisa Aaslestad, Brice Marty, Monica Patrascu, Haakon Reithe, Bettina S. Husebo, Rolf Moe Nilssen, Erika Sheard, Simon Kverneng, Charalampos Tzoulis, Line Iden Berge

**Affiliations:** 1 Centre for Elderly and Nursing Home Medicine, Department of Global Public Health and Primary Care, University of Bergen, Bergen, Norway; 2 Complex Systems Laboratory, University Politehnica of Bucharest, Bucharest, Romania; 3 Neuro-SysMed, Department of Neurology, Haukeland University Hospital, Bergen, Norway; 4 Department of Clinical Medicine, University of Bergen, Bergen, Norway; 5 K.G. Jebsen Center for Translational Research in Parkinson’s disease, University of Bergen, Bergen, Norway; 6 NKS Olaviken Gerontopsychatric Hospital, Bergen, Norway

**Keywords:** nocturnal movements, Parkinson’s disease, sensing technology, symptom assessment, wearables

## Abstract

**Background:**

Parkinson’s disease is frequently accompanied by Rapid Eye Movement (REM) sleep behavior disorder (RBD), causing individuals to physically act out their dreams. The REM Sleep Behavior Disorder Questionnaire (RBDSQ) is a 13-items self-report tool to identify individuals with probable REM Sleep Behavior Disorder (pRBD). While self-report of symptoms is limited by inaccuracies in recall and subjective interpretation, some of the RBDSQ items concerns nocturnal motor behavior that could be suitable for digital assessment. Therefore, we examined the potential of the Empatica E4 wristband to objectively support RBD assessment alongside the self-reported RBDSQ.

**Methods:**

To capture nocturnal motor behavior (e.g., number, total sleep time, magnitude) and heart rate variability, data from 149 nights were recorded continuously from 14 people with Parkinson’s disease. Data were analyzed by visual inspection, movement classification, and the Cole-Kripke algorithm. Participants also completed the RBDSQ. Cronbach’s alpha was used to determine how consistently the clinical and digital data points were measuring the same underlying construct of nocturnal motor behavior and RBDSQ defined pRBD.

**Results:**

We identified four RBDSQ items that assessed nocturnal motor behavior and there were discrepancies between self-reported RBDSQ items and sensor data for these items. Accelerometry data showed higher frequency of nocturnal motor activity in individuals with RBDSQ defined pRBD in crude models, which was not fully captured in the RBDSQ scores. We explored the potential of integrating sensor data into selected RBDSQ items, and evaluation with Cronbach’s alfa indicated high internal consistency (α = 0.87).

**Conclusion:**

Supplementing self-reported questionnaires with wearable sensor data could provide a more objective and reliable method for assessing RBDSQ defined pRBD in people with Parkinson’s disease. This approach could improve symptom assessment accuracy by reducing the subjective biases inherent in self-reported data and by capturing symptoms that are fluctuating, underreported or unrecognized.

## 1 Introduction

Due to the loss of muscle atonia, Rapid Eye Movement (REM) sleep behavior disorder (RBD) is characterized by acting out vivid dreams during REM sleep, which can lead to injuries for the individual and their bed partner (1, 2). Injuries can range from minor bruises to more serious accidents like head trauma (1, 2). RBD can also be an early indicator of α-synucleinopathies including neurodegenerative diseases such as Parkinson’s disease (PD), dementia with Lewy bodies (DLB), and multiple system atrophy (MSA) (2, 3).

According to the International Classification of Sleep Disorders criteria, a definite diagnosis of RBD requires video-polysomnography (PSG), the gold standard to confirm REM sleep without muscle atonia, involving overnight monitoring of brain activity, eye movements, and muscle activities (1). Since polysomnography is time-consuming, costly, and burdensome for the individual (3, 4), the REM Sleep Screening Questionnaire, RBDSQ (4), is often used for preliminary RBD screening in people with PD. The RBDSQ is a 13-item self-report tool including four questions concerning physical movements, nocturnal awakenings, and general sleep disturbances. While helpful for initial assessment, these items may not accurately assess the characteristic fluctuation of symptoms (5), especially in cases of mild to moderate symptoms (4). Due to potential recall bias and limited sensitivity of the tool (5, 6), there is a growing need to explore complementary objective approaches that can enhance the accuracy of RBD screening and diagnosis (7).

Wearable devices such as Actiwatch and Empatica E4 wristband have the potential to supplement RBDSQ data and to enhance the objectivity and accuracy of pRBD assessments (8). Wearables are devices worn on a person’s body, equipped with sensors that can record health data by monitoring physiological parameters such as nocturnal movement activity (7). A study by Naismith et al. used actigraphy in 23 individuals with PD and found that self-reported RBD were associated with higher numbers of wake periods compared to those without (8). In another study, Louter et al. compared actigraphy with video-polysomnography and found that actigraphy had high sensitivity but low specificity in detecting wake periods associated with RBD, suggesting overestimation of wakefulness during REM sleep (9). Moreover, heart rate variability (HRV) analysis of 26 patients with PD and 10 with idiopathic RBD revealed reduced parasympathetic modulation and diminished HRV across sleep stages, indicating autonomic dysfunction linked to RBD in a study by Bugalho et al. (10). Finally, Bhidayasiri and Mari proposed that digital phenotyping, that is, moment-by-moment quantification of individual patient phenotype with wearable sensors and mobile technology, can enhance clinical assessment by improving the accuracy, ecological validity, and patient engagement in home-based monitoring (7).

As the next step, this study aims to explore how nocturnal behavior data encompassing accelerometer and heart rate collected from the Empatica E4 wristband can be used to objectively supplement specific self-reported items from the RBDSQ in persons with PD and pRBD. We aim to address the following research questions:

1. Which RBDSQ items can be identified by face validity for digital assessment of nocturnal motor behavior?
2. What are the correlations between the sensor data features and the self-reported values on the identified RBDSQ items assessing nocturnal motor behavior?

## 2 Methods

### 2.1 Study setting and participants

Data were sourced from the DIGItal Phenotyping in people with PARKinson’s disease (DIGI. PARK) study. Participants were recruited from the STRAT-PARK cohort, a longitudinal cohort study of PD, at Neuro-SysMed, Haukeland University Hospital Norway (11). All participants had a clinical diagnosis of established or probable PD, according to the Movement Disorders Society Clinical Diagnostic Criteria (12) and a FP-CIT single photon emission CT (DaTscan) with evidence of nigrostriatal degeneration. Inclusion criteria: people with a PD diagnosis, ≥ 60 years, living at home, with a life expectancy exceeding 6 months. Exclusion criteria was comorbidity inferring with study participation. The study protocol is described in detail elsewhere (11, 13).

### 2.2 Scales and procedures

#### 2.2.1 The self-reported RBDSQ scale

Data were collected from February 2022 to December 2022, over 14 days for each study participants. In this study, we employed the RBDSQ scale (4) as the traditional outcome measure. The RBDSQ was applied at baseline (day 1), referred to as the original RBDSQ. This self-report scale encompasses 13 items of which each are scored 0 or 1 point, yielding a total score of 13 points. In line with previous studies, participants with score ≥ 5 were classified as positive for pRBD (4). The scale comprises 13 items covering dream-enacting behaviors, sleep disruption, nocturnal motor activity, and impact on bedpartners. Based on the scores on the original RBDSQ, participants in this study were classified as having pRBD (RBDSQ defined pRBD), indicating a high likelihood of experiencing RBD symptoms, and as non-RBD, indicating no significant RBD symptoms (4).

#### 2.2.2 The selection of RBDSQ items for digital mapping

To incorporate objective data into the original RBDSQ, we selected specific items indicative of physical movements during sleep. Based on face validity, we chose items 4 (*I know that my arms or legs move when I sleep*), 6.2 (*I have or had the following phenomena during my dreams: sudden limb movements, fights*), 7 (*It happens that my movements awake me*), and 9 (*My sleep is frequently disturbed*), as these items involve reports of physical movement which could be suitable for objective measurement using the Empatica E4 wristband. To explore the potential for digitally supplementing the RBDSQ, we replaced the original self-reported responses to the items (4, 6.2, 7, and 9) with objective data derived from wearable sensors. The remainign items (1–3, 5, 6.1, 6.3, 6.4, 8, and 10) were retained in their original self-reported format. Together, these formed the digital RBDSQ (D-RBDSQ); a composite scale combining subjective and objective data to assess symptoms of pRBD in individuals with PD.

#### 2.2.3 The digital assessment with Empatica E4 wristband

As a digital measure we applied the Empatica E4 wristband (14), which was worn continuously by each participant on the left wrist for 7 days before switching to the right wrist for another 7 days, with the aim of capturing PD symptom asymmetry. The watch is considered a medical research-grade device (CE Crt. No. 1876/MDD, 93/42/EEC). It’s used by researchers to collect physiological data in various settings, including real-world environments. It has the ability to measure and stream real-time data, including heart rate, electrodermal activity (EDA), temperature, and motion. In this study, we used data from the devices accelerometer and photoplethysmography (PPG) sensors. The accelerometer measures the continuous gravitational force along the x, y, and z-axes within a range of (−2 g, 2 g) at the sampling frequency of 32 Hz. The PPG sensor records heart rate variability (HRV), providing information about the inter-beat interval (IBI), and allowing for the calculation of the heart rate (HR) at the sampling rate of 64 Hz.

Data were analyzed using proprietary software to identify disturbances and classify sleep patterns. These analyses were used to generate values for items 4, 6.2, 7, and 9 in the original RBDSQ, forming the basis for their integration into the digitally supplemented D-RBDSQ scale.

### 2.3 Statistical and digital analyses

#### 2.3.1 Statistical analyses of group differences

The Kolmogorov–Smirnov test suggested non-normal distribution of all continuous variables. Therefore, we employed the Mann–Whitney U test for comparing independent samples to evaluate differences between the participants with and without RBDSQ defined pRBD. Chi-square test was used to evaluate group differences and compare the proportion of heart rate arousals related to wake periods between RBDSQ defined pRBDvs. nRBD nights. Sleep parameters derived from accelerometry, movement classification, and heart rate variability were summarized for both groups, and group comparisons were conducted across these domains. We used mixed linear regression models to evaluate differences in the continuous outcome variables between the participants with and without RBDSQ defined pRBD while also adjusting for age and MDS-UPDRS III in two separate models. We did not correct the *p*-values for multiple testing, as our analyses were exploratory with the aim of generating hypotheses rather than confirming predefined hypotheses. The two-proportion z-test was applied for comparing proportions of positive items of the sensor data derived items (4, 6.2, 7, and 9) from the D-RBDSQ scale against positive items on the original RBDSQ scale.

#### 2.3.2 Psychometric evaluation of the RBDSQ and D-RBDSQ

We used Cronbach’s alpha to evaluate the reliability in the original RBDSQ and the D-RBDSQ scales. Corrected Item-Total Correlation measures the correlation of each item with the total score, excluding the item itself, helping to identify items that do not correlate well with the overall scale. Cronbach’s alpha if Item Deleted indicates how the overall reliability would change if a particular item were removed from the scale, providing insight into the contribution of each item to the overall consistency. Results were considered significant for *p* < 0.05.

#### 2.3.3 Preprocessing and visualization of wearable data

**Figure 1:**
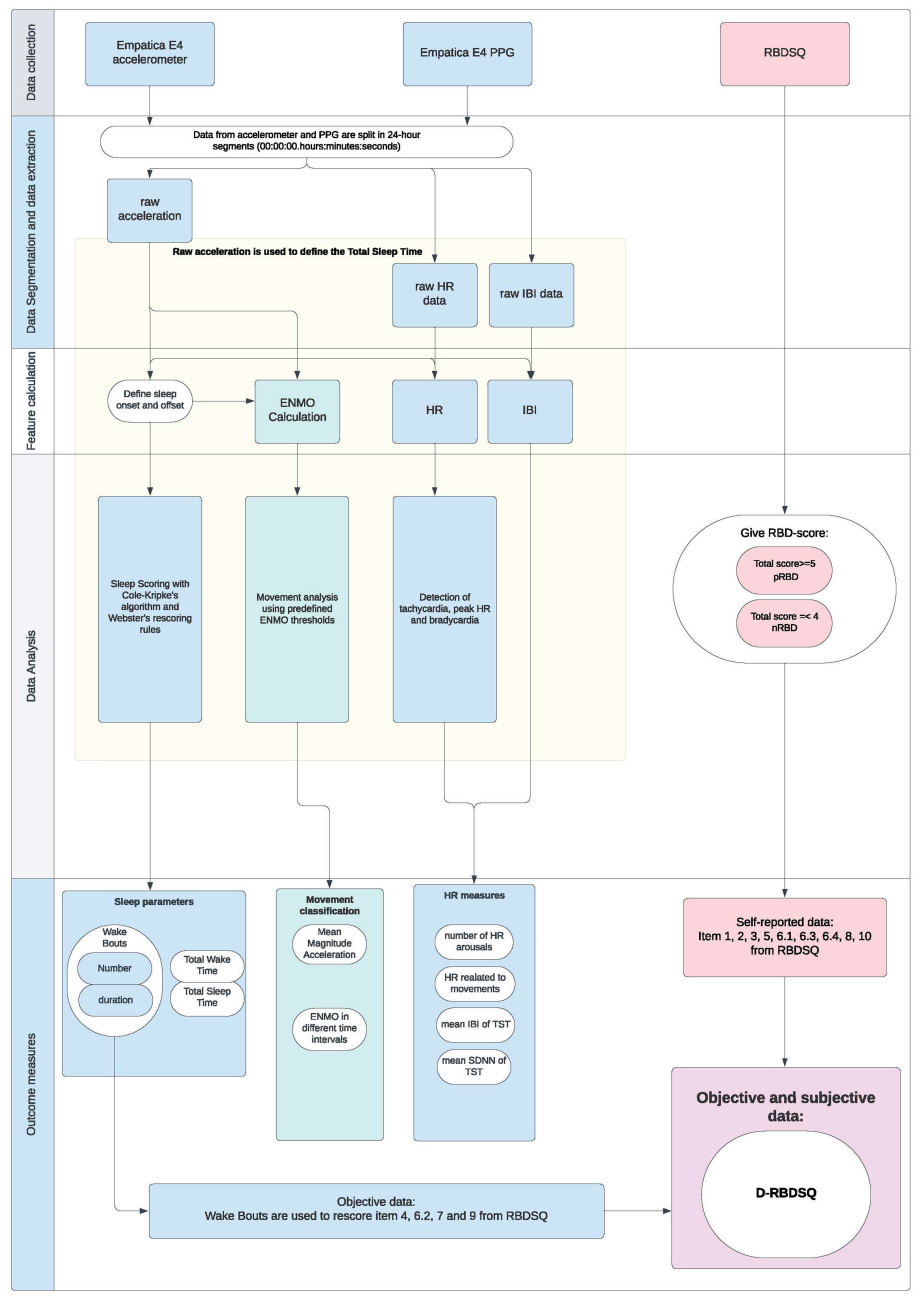
Data processing and analysis flowchart for the development of D-RBDSQ. PPG, Photoplethysmography; RBDSQ, REM Sleep Behavior Disorder Questionnaire; HR, Heart Rate; IBI, Inter-Beat Interval; ENMO, Euclidean Norm Minus One; RBD, REM Sleep Behavior Disorder; pRBD, Probable RBD; nRBD, No RBD; TST, Total Sleep Time; SDNN, Standard Deviation of the NN Interval (in this case, the IBI); D-RBDSQ, Digital RBDSQ.

An overview of data processing and analysis flowchart for the integration of sensor data into the D-RBDSQ is provided in Figure 1.

Data from accelerometry, HRV (IBI), and HR were split into 24-h segments for day-to-day analyses. Visualizations of these data segments were carried out using MATLAB version 2023a. Visualizations of time-series helped identify trends and anomalies in sleep patterns, HRV, and nocturnal motor activity during sleep.

#### 2.3.4 Sleep scoring and analyses of nocturnal behavior

##### 2.3.4.1 Definition of sleep window and sleep scoring

We analyzed activity and inactivity through visual inspection of each 24-h segment within a predefined sleep window (23:00–08:00). The sleep window was heuristically established, reflecting the typical sleep schedule of the general population (15). The total sleep time (TST) was defined within this timeframe as the presence of 20 consecutive minutes of significantly reduced activity (16). The sleep period ended when there was a noticeable increase in activity, indicating the participant had likely left the bed. In this study, the terms ‘sleep’ and ‘sleep disturbance’ refer to periods of reduced activity during night-time and the interruptions in these periods, respectively.

During the TST, we identified movements by manually marking noticeable spikes in acceleration data, each labelled as a ‘movement’. After cataloguing of all movements, we conducted two separate analyses: 1) analyzing sleep features using the Cole-Kripke sleep-scoring algorithm (17) with sleep rescoring-rules by Webster et al. (18) to extract sleep features such as wake periods, their duration, and total wake time; 2) detection of significant nocturnal behaviors, covering the characteristics of movements, their frequency, duration, intensity, and overall acceleration throughout the TST.

The sleep scoring rules by Cole-Kripke (17) and Webster et al. (18) has been validated against video-PSG (16). Wakefulness was determined by setting a threshold at 100 m/s^2^ to initiate a ‘wake period’ in these analyses.

#### 2.3.5 Feature extraction from accelerometry data

Movement characteristics were estimated with the Euclidean Norm Minus One (ENMO) from the body acceleration alongside all triaxial acceleration, known as the vector magnitude (VM):


$$\mathrm{VM}=\sqrt{\left(x^{2}+y^{2}+z^{2}\right)}$$

where x, y and z are the accelerations along three orthogonal axes, respectively.

The calculation of ENMO involves adjusting the VM for gravity by subtracting a fixed value of 64 (equivalent to 1 g, with 1 g = 9.81 m/s^2^ as described in the Empatica E4 manual (14)) from the VM as per the equation:


ENMO=VM−64

The ENMO specifically isolates dynamic acceleration by subtracting one unit of gravitational force from the VM and setting negative values to zero, expressed in milli gravitational units (mg), with 1,000 mg corresponding to the force of gravity near the Earth surface. This method ensures that the measurement emphasizes movement-related acceleration, free from static gravitational effects (19).

To classify nocturnal activities, we applied ENMO threshold values identified by Bakrania et al. (19) and Hildebrand et al. (20). Thus, we classified isolated movements based on their duration and acceleration, employing threshold values from Bakrania et al. (19), to distinguish between sedentary behaviors and light-intensity physical activities. Although the demographic profile of these studies does not directly match our study participants, the thresholds serve a basis for identifying sedentary versus light activity levels. Additionally, we opted for a conservative threshold of 8.7 mg. to differentiate between sleep and wake, and a higher threshold of 21.6 for seperating smaller and larger movements, as suggested by Bakrania et al. (19) and Hildebrand et al. (20).

#### 2.3.6 HR and HRV as indicators of nocturnal arousals

This study employed two cardiovascular measures: HR and HRV, which were analyzed throughout the TST to explore the autonomic nervous systems response to nocturnal body movements. HR changes related to physical arousals are typically characterized by an increase (tachycardia) followed by a decrease (bradycardia) (21). The severity of the arousal is strongly associated with tachycardia (21).

HR changes, including the start of tachycardia, peak HR, and the onset of bradycardia, were manually identified, and documented. The time stamps of these changes were compared to the corresponding time stamp in the accelerometery data. If tachycardia and movement occurred together, or if tachycardia started up to 2 s before movement, they were classified as related (21).

For HRV analyses, we calculated the mean beat-to-beat heart rate variations, referred to as the IBI, and the IBI standard deviation (SDNN) across the TST.

#### 2.3.7 Integration of objective metrics into selected RBDSQ items

To accurately capture events of relevant movements, we proposed a series of threshold values for each of items 4, 6.2, 7, and 9 in the RBDSQ. We then recalculated the scores for these items based on average movement features, sampled from all recorded nights, for each participant. The criteria used to interpret these items based on accelerometery data were:

- *RBDSQ 4. I know that my arms or legs move when I sleep:* Score ‘yes’ if the movements ENMO ≥ 8.7 mg (19) and duration ≥ 5 min.

If not, score the item as ‘no’.

- *RBDSQ 6.2. I have or had the following phenomena during my dreams: sudden limb movements, fights:* Score ‘yes’ if the average number of wake periods ≥ 8 (22) per night and if any movements ENMO ≥ 21.6 mg (20).

If not, score the item as ‘no’.

- *RBDSQ 7. It happens that my movements awake me:* Score yes if number of wake periods ≥ 8 (22).

If not, score the item as ‘no’.

- *RBDSQ 9*. *My sleep is frequently disturbed:* Score yes if number of wake periods ≥ 8 (22) in over 50% of measured nights.

If not, score the item as ‘no’.

Following this method, the scores of the revised items (4, 6.2, 7, and 9) together with the scores of the self-reported items (item 1–3, 5, 6.1, 6.3, 8, and 10) are referred to as the D-RBDSQ.

## 3 Results

A total of 14 participants were included in the study. Sensor data was obtained from a total of 148 nights from 14 participants in this study. Data from 11 of these nights were incompletely recorded and therefore excluded from further analyses. Of these, 8 nights had incomplete measurements due to the device being removed by the participant, 2 nights had data that could not be read due to device errors, and 1night lost contact with the participant in the middle of the sleep period. Additionally, 7 of these nights were excluded from the cardiovascular analyses because the PPG sensor stops recording during periods of significant wrist movement. After excluding incomplete and failed recordings, 137 nights were included in the accelerometry analyses and 130 nights were included in the heart rate analyses.

### 3.1 Descriptive clinical and accelerometry data

Based on the self-reported RBD scores, a total of 5 participants were classified as pRBD (RBDSQ defined pRBD), while 9 participants were classified as without (nRBD). A comparison of clinical data and sensor-based data between the RBDSQ defined pRBD and nRBD group is presented in Table 1.

**Table 1:** Descriptive clinical and sensor data for RBDSQ defined pRBD and nRBD.

Characteristics	RBDSQ defined pRBD (*n*)	nRBD (*n*)	Total	*p-*Values
Participants^#^	5	9	14	0.29
Age, median (SD)	80 (7.68)	68 (5.40)		**0.03**
Hoehn and Yahr stage (SD)	2 (0)	1.87 (0.33)		0.53
MDS-UPDRS III	39.20 (15.34)	26.62 (10.38)		0.05
Accelerometry recordings				
N measured nights	52	96	148	0.98
Measured nights per participant, mean (SD)	10.40 (2.41)	10.66 (1.66)		0.99
Recorded time, minutes	468	864	1,332	0.14
Heart rate variability recordings				
Total nights	49	92	141	0.45
Total data points/ patient, mean (SD)	17,501 (6045)	18,028 (7206)	1,746,168	0.47

Most *p-*values calculated using Mann-Whitey U-test. *p*-values from the Chi-square test are indicated by a hashtag (#). Bold *p*-values indicate statistical significance (p < 0.05). SD = standard deviation. Hoehn and Yahr stage, scale-range 1–5, high score indicates greater severity of symptoms. Movement Disorder Society (MDS) Unified Parkinson’s Disease Rating Scale (UPDRS) Modified Unified Parkinson’s Disease Rating Scale (MDS-UPDRS) Part III (motor examination), scale range 0–132, high score indicates greater severity of motor symptoms.

The average age of participants with RBDSQ defined pRBD was higher compared to those without, with mean ages of 80 and 68 years, respectively. No relevant differences in accelerometry and heart rate data from Empatica E4 wristband were observed between the groups, demonstrating a balanced distribution of measured data points, number of nights measured and recorded time.

### 3.2 Sleep parameters, movement classification and heart rate measures derived from accelerometer data

Participants with RBDSQ defined pRBD showed more fragmented sleep compared to those without in crude models. Specifically, RBDSQ defined pRBD nights had a higher number of total wake periods (mean = 12.46, SD = 5.58 vs. 9.80, SD = 5.52; *p* = 0.002), longer average duration of wake periods per night (mean = 19.89 min, SD = 26.31 vs. 9.80 min, SD = 5.52; *p* = 0.005), and greater total wake time (mean = 57.71 min, SD = 50.49 vs. 23.66 min, SD = 36.61; *p* < 0.001). Additionally, the number of wake periods lasting at least 5 min and the average duration of these awakenings were significantly greater in pRBD nights (mean = 2.71, SD = 1.68 vs. 1.15, SD = 1.17; *p* = 0.009) in crude models (Table 2).

**Table 2:** Sleep parameters during total sleep time (TST).

Characteristics	RBDSQ defined pRBD mean (standard deviation)	nRBD mean (standard deviation)	*p* values
Accelerometry			
Total sleep time, min	434.23 (48.36)	440.71 (56.07)	0.14
N Wake periods,	12.46 (5.58)	9.80 (5.52)	**0.002**
Duration, min	19.89 (26.31)	9.80 (5.52)	**0.005**
Wake periods ≥ 5 min, number	2.71 (1.68)	1.15 (1.17)	**0.009**
Total wake time, minutes	57.71 (50.49)	23.66 (36.61)	**0.00000000036**
Movement classification during total sleep time (TST)			
Mean magnitude acceleration, mg	5.11 (2.54)	4.26 (3.14)	0.03
ENMO threshold ≥ 8.7, duration ≥ 5 s			
N movements	16.10 (10.41)	11.60 (7.19)	0.06
Mean magnitude acceleration, mg	21.74 (6.68)	22.99 (6.72)	0.34
ENMO threshold ≥ 8.7 mg, duration ≥ 25 s			
N movements	10.08 (4.61)	5.66 (4.20)	**0.004**
Mean magnitude acceleration, mg	20.63(6.02)	21.81 (8.34)	0.47
ENMO threshold ≥ 8.7 mg, duration ≥ 5 min			
N movements	20.40 (9.91)	8.22 (7.72)	0.06
Mean magnitude acceleration, mg	22.07 (6.38)	16.36 (10.26)	0.44
ENMO threshold ≥ 21.6 mg, duration ≥ 5 s			
N movements	14.80 (6.07)	7.03 (3.88)	0.46
Mean magnitude acceleration, mg	31.59 (3.65)	31.96 (3.79)	**0.89**
ENMO threshold ≥ 21.6 mg, duration ≥ 25 s			
N movements	7.24 (4.43)	3.69 (1.72)	0.20
Mean magnitude acceleration, mg	33.30 (5.66)	32.72 (5.64)	0.89
ENMO threshold ≥ 21.6 mg, duration ≥ 5 min			
N movements	9.40 (4.72)	4.22 (3.99)	0.06
Mean magnitude acceleration, mg	27.73 (3.89)	21.00 (13.05)	0.14
Heart rate measures			
N HR arousals	7.44 (1.63)	6.60 (0.65)	0.07
HR arousals related to wake periods, percent^#^	68.99 (20.72)	54.97 (30.39)	0.37
IBI, ms	984.36 (224.37)	891.98 (135.13)	0.95
SDNN, ms	72.51 (25.48)	86.73 (21.18)	0.19

Most *p-*values calculated using Mann-Whitey U-test. *p*-values from the Chi-square test are indicated by a hashtag (^#^). *p*-values are crude, i.e., Not adjusted for age and MDP-UPDRSIII. Bold *p*-values indicate statistical significance (*p* < 0.05). ENMO, Euclidean Norm Minus One (measures in mg milligravity units); mg, milligravity units; IBI, Inter beat interval; SDNN, Standard deviation of the inter beat interval; ms, milliseconds.

Furthermore, RBDSQ defined pRBD nights were characterized by a significantly higher overall mean magnitude of acceleration during total sleep time (mean = 5.11 mg, SD = 2.54 vs. 4.26 mg, SD = 3.14; *p* = 0.03), and a greater number of movements exceeding 8.7 mg in ENMO lasting at least 25 s (mean = 10.08, SD = 4.61 vs. 5.66, SD = 4.20; *p* = 0.004) in crude models. No other significant differences were observed in movement characteristics across the various ENMO thresholds and durations. When adjusting these analyses for age and MDS-UPDRSIII score in two separate models, none of the differences between RBDSQ defined pRBD and nRBD remained significant in either model (data not shown).

There were no statistically significant differences in heart rate measures during nighttime between RBDSQ defined pRBD and nRBD nights, both in crude and age and MDS-UPDRSIII adjusted models.

To assess internal consistency, we calculated Cronbach’s alpha for both the original RBDSQ scale, which summarizes the scores on the 13 self-reported items, and the D-RBDSQ scale summarizing the scores on the 4 objective and the 9 self-reported items. As shown in Table 3, the original RBDSQ demonstrated higher internal consistency (Cronbach’s *α* = 0.92) compared to the D-RBDSQ (α = 0.84). In the D-RBDSQ, item 6.2 (“I have had the following phenomena during my dreams: sudden limb movements, fights”) showed a negative corrected item-total correlation and an increase in Cronbach’s alpha to 0.87 if the item was deleted. The remaining items contributed positively to internal consistency.

**Table 3:** Cronbach’s alpha on RBDSQ and D-RBDSQ.

RBDSQ item (question)	RBDSQ			D-RBDSQ			
	Mean	Corrected item-total correlation	Cronbach’s alpha if item deleted	Mean	Corrected item-total correlation	Cronbach’s alpha if item deleted	Mean
Q 1	0.64	0.49	0.91	0.64	0.35	0.84	0.64
Q 2	0.21	0.86	0.90	0.21	0.73	0.81	0.21
Q 3	0.21	0.26	0.92	0.21	0.32	0.84	0.21
**Q 4**	**0.43**	**0.80**	**0.90**	**0.57**	**0.67**	**0.81**	**0.57**
Q 5	0.29	0.85	0.90	0.29	0.75	0.81	0.29
Q 6.1	0.29	0.85	0.90	0.29	0.75	0.81	0.29
**Q 6.2**	**0.29**	**0.85**	**0.90**	**0.50**	**−0.13**	**0.87**	**0.50**
Q 6.3	0.21	0.86	0.90	0.21	0.73	0.81	0.21
Q 6.4	0.29	0.85	0.90	0.36	0.59	0.82	0.36
**Q 7**	**0.29**	**0.71**	**0.91**	**0.57**	**0.67**	**0.81**	**0.57**
Q 8	0.43	0.07	0.931	0.43	0.18	0.85	
**Q 9**	**0.36**	**0.75**	**0.90**	**0.64**	**0.76**	**0.81**	
Q 10	1	0.00	0.92	1	0.00	0.85	
Average unweighted Cronbach’s Alpha			0.92			0.84	

Bold numbers refer to the RBDSQ items that were rescored (item 4, 6.2, 7, 9).

In the D-RBDSQ, item 6.2 (‘*I have had the following phenomena during my dreams: sudden limb movements, fights)* showed a negative corrected item-total correlation and increased Cronbach’s alpha from 0.84 to 0.87 when removed. The remaining items contributed positively to internal consistency.

A two-proportion z-test was conducted to compare positive responses on the four items (4, 6.2, 7, 9) summarized that were rescored using sensor-based data in the D-RBDSQ with the original RBDSQ. This yielded z = −2.27 and *p* = 0.02, which indicates statistically significant difference. Additionally, a comparison was made between the total scores of the D-RBDSQ and the original RBDSQ showed no statistically significant difference (z = −1.28 and *p* = 0.20).

## 4 Discussion

This study explores the integration of sensor data into the original RBDSQ, to support a more objective digital assessment of pRBD in people with PD. Utilizing Empatica E4, we found a difference between participants with and without RBDSQ defined pRBD in crude analysis, particularly for longer and more intense movements during nocturnal wake periods. Meanwhile, no differences were found in heart rate variability between participants with and without RBDSQ defined pRBD. A high Cronbach’s alpha demonstrated that the self-reported clinical and digital data measured the same underlying construct of nocturnal motor behavior and pRBD. Results are of key importance for people with PD, their relatives, and clinicians because they highlight the limitations of self-reported symptom assessment and illustrate the potential for supplementing validated assessment tools such as the RBDSQ with objective data to support a more reliable assessment of pRBD.

The sensor data indicated that participants with RBDSQ defined pRBD experienced a higher mean number of wake periods with longer duration and more minutes of wakefulness after sleep onset each night, compared to those without RBDSQ defined pRBD in crude analyses. This is in line with previous studies demonstrating an increased proportion of wake periods among participants with RBD (8, 9, 23). Accelerometry has demonstrated high specificity (95%) but low sensitivity (20%) for detecting wake periods in elderly individuals with PD. (24) This discrepancy may explain why some studies report shorter wake periods in PD with RBD (9), while others suggest longer wake periods compared to controls (24). The lack of a standardized methodology for accelerometry based sleep/wake detection in people with PD and RBD likely contributes to this variation (8). We aimed to minimize misclassification risk due to incorrect scoring by applying the Cole-Kripke algorithm with rescoring rules by Webster et al. (18). These rules consolidate fragmented events of increased activity into a single period of wakefulness if they meet specific criteria (7). Although this approach may lead to perceiving each wake period as longer, the total wake duration during total sleep time (TST) serves as a more robust indicator of nocturnal motor activity.

Participants with RBDSQ defined pRBD exhibited an overall increase in the number of movements across all categories, compared to participants without RBDSQ defined pRBD. However, statistically significant differences between groups were only observed for movements exceeding 8.7 mg in acceleration and lasting more than 25 s in crude models. This suggests that while overall movement may be elevated in RBD, only specific patterns of sustained and higher-intensity movement differentiate the groups. These findings may be influenced by the exploratory nature of our method, as applied threshold from the existing literature were developed for daytime behaviors that may not adequately reflect the characteristics of nocturnal movement (19, 20). Due to the lack of established thresholds for nocturnal behaviors, we used sedentary behavior thresholds to classify sleep-related activities. We suggest that future studies should develop and test sensor thresholds for nocturnal behaviors in this population.

We did not find significant differences in mean NN intervals and SDNN between participants with or without RBDSQ defined pRBD. Previous studies have shown that PD patients with RBD exhibit blunted HRV in different sleep stages compared to PD patients without RBD (10). In our study, when HRV measures were assessed over the TST, differences between participants with and without RBDSQ defined pRBD were no longer significant (10). Additionally, the PPG sensor in the Empatica E4 stops recording during large wrist movements, which could contribute to our finding of no difference in HR and HRV between those with and without RBDSQ defined pRBD. As such, we did not include HRV measures in our sleep/ wake analyses. However, both PD patients with and without RBD can have autonomic dysfunction (25), and it is currently discussed whether this dysfunction is more severe or presents differently in those with RBD (23).

We selected the RBDSQ items capturing movements that we *a priori* expected to be measurable by accelerometry. Analyses of the D-RBDSQ item 6.2 (‘*I have had the following phenomena during my dreams: sudden limb movements, fights*) however showed a negative corrected item-total correlation and increased Cronbach’s alpha when removed, which could suggest that it is challenging to assess presence of dreaming and dream behavior with a wearable. Unlike previous studies that apply machine learning algorithms from wearables (8, 26), our method integrates sensor-based data directly into the original RBDSQ. This integration is promising in making self-reported questionnaires more objective but requires careful implementation to avoid redundancy and to ensure that each item distinctly measures the symptom/behavior under investigation. Applying this method, we found statistically significant differences in response proportions between the original RBDSQ and the D-RBDSQ for the selected items (items 4, 6.2, 7, and 9). The negative z-value indicates that responses for these items were less frequently reported as “yes” in the original RBDSQ compared to the D-RBDSQ. However, when considering all 13 items across both questionnaires, there were no significant differences in response proportions, suggesting that the essential psychometric properties of the D-RBDSQ are maintained when incorporating sensor-based data.

Our study has limitations. Despite collecting extensive data on each participant, the low number of participants limits the generalizability of our findings. While the study aimed to explore the potential for developing a digital version of the RBDSQ (D-RBDSQ), the small number of participants with RBDSQ defined pRBD limits the possibility of validating the new scale in a different sample than it was developed on. We explicitly highlight that the definitive diagnosis of RBD via PSG was not available; classification was therefore based on the RBDSQ, classifying participants as RBDSQ defined probable or non-probable RBD. Further, while accelerometry provides valuable insights into sleep patterns by measuring activity levels, it is not intended to replace PSG. Instead, to enhance the screening utility of the RBDSQ for pRBD, the modified D-RBDSQ is designed to incorporate a set of objective measures. Finally, participants with RBDSQ defined pRBD were significantly older than those without (80 years vs. 68 years), which explained the statistically significant differences between the groups on all outcomes in the crude models, and we suggest that the results from the crude models should be considered exploratory rather than confirmatory. x.

## 5 Conclusion

Our findings suggest that wearable sensor technology can provide critical insights into pRBD sleep patterns in people with PD. By supplementing self-reported questionnaire items with accelerometry data from Empatica E4 wristband, we revealed nocturnal motor behavior indicative of RBDSQ defined pRBD which were not as evident through self-reported questionnaire data alone. Our findings support the feasibility of detecting RBDSQ defined pRBD-related motor behaviors using wearable accelerometry and suggest that specific movement characteristics, particularly those of higher intensity and longer duration, may serve as digital correlates of pRBD symptoms. Given that people with PD often have cognitive impairment and that traditional questionnaires rely on self-report or bed partner observations, supplementation with digital data may improve the reliability and clinical utility of pRBD screening. Considering the adverse impact of RBD on PD progression, larger studies are warranted to refine sensor thresholds for nocturnal behaviors, validate digital scoring algorithms, and assess clinical applicability across diverse populations. In particular, studies should explore the diagnostic accuracy of the D-RBDSQ against PSG for people with pRBD. Ultimately, this approach may contribute to more equitable, scalable, and objective screening for pRBD, both in the inpatient and home-based settings.

## Data Availability

The datasets presented in this article are not readily available because the datasets used in the current study are protected under privacy regulations. Datasets can be made available in deidentified form by reasonable request to the corresponding author. Requests to access the datasets should be directed to Lisa Aaslestad, lisa.aaslestad@uib.no.
